# Alvis: a tool for contig and read ALignment VISualisation and chimera detection

**DOI:** 10.1186/s12859-021-04056-0

**Published:** 2021-03-16

**Authors:** Samuel Martin, Richard M. Leggett

**Affiliations:** Earlham Institute, Norwich Research Park, Norwich, UK

**Keywords:** Sequence alignment, Genomics, Visualisation, Chimera detection

## Abstract

**Background:**

The analysis of long reads or the assessment of assembly or target capture data often necessitates running alignments against reference genomes or gene sets. The aligner outputs are often parsed automatically by scripts, but many kinds of analysis can benefit from the understanding that can follow human inspection of individual alignments. Additionally, diagrams are a useful means of communicating assembly results to others.

**Results:**

We developed Alvis, a simple command line tool that can generate visualisations for a number of common alignment analysis tasks. Alvis is a fast and portable tool that accepts input in a variety of alignment formats and will output production ready vector images. Additionally, Alvis will highlight potentially chimeric reads or contigs, a common source of misassemblies.

**Conclusion:**

Alvis diagrams facilitate improved understanding of assembly quality, enable read coverage to be visualised and potential errors to be identified. Additionally, we found that splitting chimeric reads using the output provided by Alvis can improve the contiguity of assemblies, while maintaining correctness.

## Background

Finding alignments between two sets of sequences is a fundamental task in bioinformatics. In particular, the analysis of long reads, the assessment of assembly results or evaluation of target capture protocols often necessitate alignment against reference genomes or gene sets. Many different tools exist to calculate alignments and these tools generate a range of different output formats. Most of the common formats consist of large tab separated lists which are designed for easy computer parsing rather than to convey intuitive human understanding. Yet many kinds of analysis can benefit from a visual depiction of alignments—for example, inspecting the layout of contigs across a chromosome, understanding where assemblies break down or picturing read coverage depth across a chromosome or gene. Such analysis can also expose the presence of chimeras formed by the artificial joining of disconnected parts of the genome.

### Existing visualisation tools

There are a number of existing tools that incorporate some form of visualisation of alignment data. One option is to use a genome browser, such as Ensembl [1], Artemis [2], or Geneious. Genome browsers are typically designed to perform much more than just sequence alignment visualisation, allowing the user to align, browse, search and analyse genomic sequence and annotation data arranged along genome tracks. Some of these tools, such as Artemis, also produce pile-up and coverage plots of reads against a reference genome. Genome browsers provide an interactive graphical interface to users and many are available online through a web browser, which can be helpful for those not familiar with the command line. However, the wide range of tasks possible in a genome browser necessitates some complexity in the user interface and the images they produce are not generally designed for publication. Additionally, many genome browsers are designed to highlight genomic features and are not best suited for understanding assembly completeness or structural problems.

Two popular tools to visualise whole genome alignments, and in particular, structural rearrangements, are Mauve [3], and Icarus [4] (part of the QUAST [5] package). Both of these tools employ their own alignment algorithms that are designed to be used in specific circumstances; Mauve performs multiple genome alignment to highlight large rearrangement events, while QUAST uses nucmer [6] alignments to assess assemblies against a reference genome. This makes them unsuitable for visualising alignments for other common tasks, such as aligning reads to a reference.

The Circos software [7] is a popular tool for producing high quality images for publication, particularly for displaying attributes related to genome position (see e.g. [8]), and for structural variation between genomes. However, while these diagrams look attractive, they can quickly become busy and are not best suited to displaying individual alignments in a meaningful manner.

The Mummerplot program, included in the MUMmer suite [6], can create a dot plot of alignments between two sets of sequences. These plots are simple, clear and easy to read, and the program itself has a simple command line interface which is easy to use. They do however, require the user to use the alignment programs included in the MUMmer package, and can become difficult to read if there are many query or target sequences, so are best suited to whole genome alignment, rather than read to genome alignment.

**Fig. 1:**
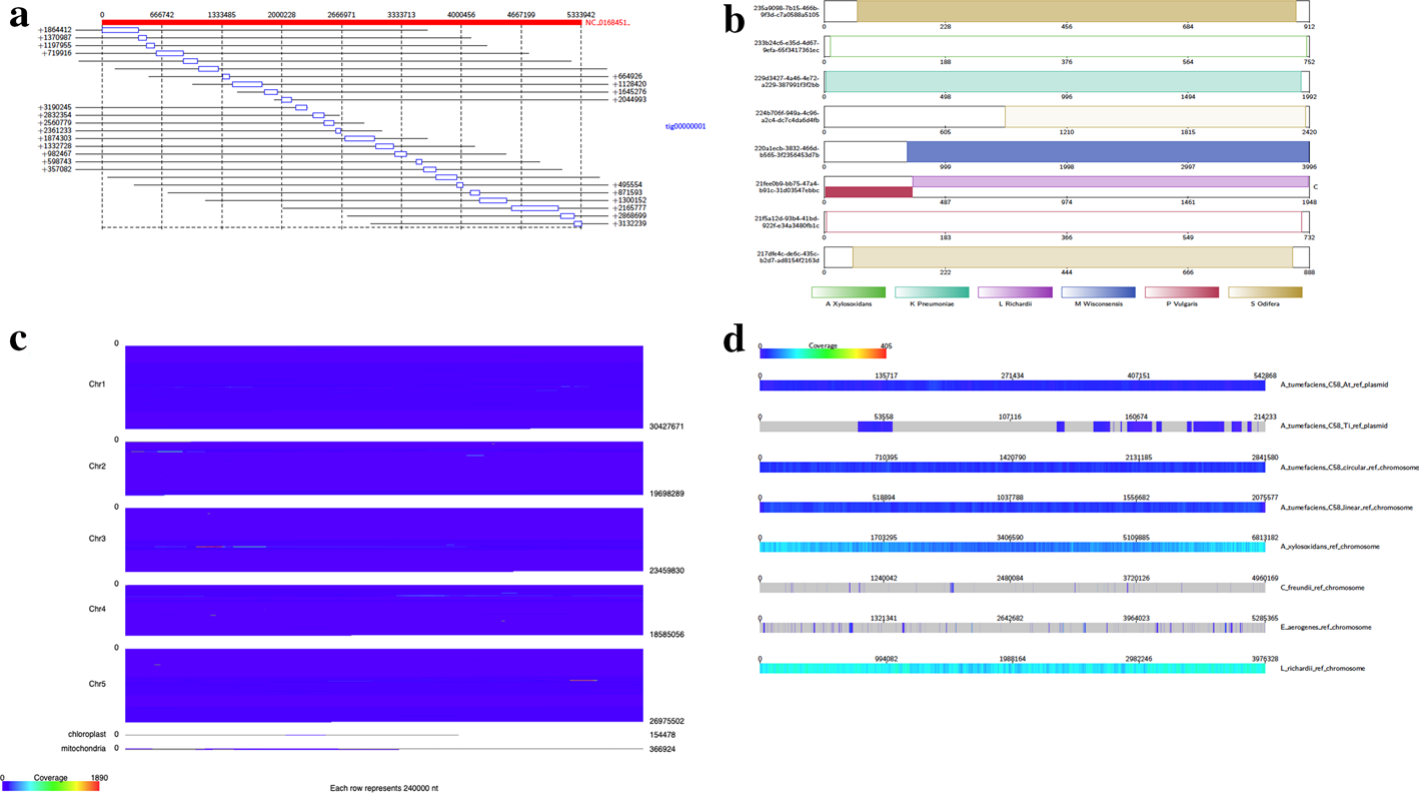
Example Alvis outputs: **a** Alignment diagram showing mapping of a contig against a reference genome. **b** Contig alignment diagram indicating a potential chimera in one of the reads. **c** Genome coverage diagram showing reading coverages across *Arabidopsis thaliana* chromosomes. **d** Coverage map showing read coverage across a set of references. Figure generated with Alvis v1.1

For aligning individual reads to a reference, one option is to use BLAST’s web interface [9]. This allows the user to enter a sequence, which is queried against NCBI databases using BLAST. Alignments to the best hit are displayed on a diagram (similar to that in Fig. 1a). This is a popular method for aligning reads against databases, however, it is unsuitable for analysing large numbers of sequences, comparing genome-scale alignments, or for comparing several alignments at once.

### Nanopore reads

The emergence of nanopore sequencing technology has allowed users to sequence reads much longer than previously possible, enabled real time analysis of sequencing data, and introduced the possibility of in-field sequencing [10]. The length of nanopore reads in particular is proving to be attractive, allowing researchers to resolve repeat regions and fill gaps in assemblies. Nanopore sequence data has a higher error rate compared to earlier technologies, and the nature of the errors is also different, with a higher prevalence of insertions and deletions. This difference in error profile has necessitated the development of new tools to perform analysis specifically for nanopore reads. One of the most popular new tools is the aligner minimap2 [11], which has its own alignment format, PAF (pairwise alignment format). None of the visualisation tools described can accept this format.

A key task when assessing nanopore reads is the detection of chimeras. Chimeric reads can be formed by the ligation of two distinct molecules during library preparation, or formed *in silico* by the base calling software when two molecules are sequenced in the same pore in short succession. A recent study found that at least 1.7% of nanopore reads contain post-amplification chimeric elements [12]. Several tools exist for the detection of chimeric nanopore reads, such as MiniScrub [13] and YACRD [14]. MiniScrub performs ‘read scrubbing’, the process of removing low quality segments in reads, which often includes chimeras, with the aim of improving the accuracy of downstream analyses. YACRD is a standalone tool for detecting chimeras in nanopore reads, and reporting these to the user. Both of these tools use a *de-novo* approach by requiring an alignment file of overlaps between reads. This has the advantage of not requiring a reference sequence, but in practice requires very high coverage to be effective, making it unsuitable for many applications e.g. metagenomics.

The problem of chimeric reads is not unique to nanopore technology, and many tools exist for the detection of chimeric 16S and ITU sequences in ecological studies, where it is essential to avoid inferring chimeric reads as new species. Popular examples include UCHIME [15] and ChimeraSlayer [16]. These tools require a high quality curated database of 16S or ITU sequences, or rely on relative abundances to perform *de-novo* detection. Whilst, in principle, it may be possible to adapt these methods for nanopore reads, little work has been done and it is unclear how well the methods will perform on comparatively noisy nanopore reads.

Here we present Alvis, a tool for visualising alignments of long reads and assemblies which can generate four different types of publication quality diagram from a wide range of different input file types. Significantly, Alvis supports the PAF format from minimap2. Alvis enables flexible filtering of alignments by the user and will automatically highlight potentially chimeric reads or contigs. Furthermore, the user may choose to view only potentially chimeric sequences and obtain a text file containing a list of all these sequences and the approximate join location. We show that applying this option to long read sequence data can improve the contiguity of assemblies. Whilst the alignment diagrams have been designed specifically to visualise alignments of long reads, visualisation of assemblies against references is not restricted to any read type, and the coverage diagrams can be used to display read coverage for any read type. Indeed, the only restriction is the format of the alignment, not the data used to generate it.

Table 1 provides an overview of the features of Alvis compared to other alignment visualisation software.

**Table 1:** Overview of tools for visualising alignments

	Genome alignment	Read alignment	Input formats	Interactive viewer	Output formats	Platforms supported
Alvis	Yes	Yes	BLAST, MUMmer, SAM, PAF, PSL	No	TeX(PDF), SVG	Java (Linux, Mac, Windows)
Artemis	Yes	Yes	BAM	Yes	–	Java 9 only
BLAST Web	No	Yes	–	No	Webpage	Browser based
Circos	Yes	Yes	Bespoke format	No	PNG, SVG	Linux, Mac, Windows
Icarus	Yes	No	–	Yes	–	Linux, Mac
Mauve	Yes	No	–	Yes	PDF	Linux, Mac, Windows
MUMmerplot	Yes	No	MUMmer	No	Postscript, X11	Linux, Mac

The Input Formats field is left empty when the visualisation is part of an alignment pipeline. The Output Formats field is left empty if the visualisation appears in an interactive display

## Implementation

Alvis is written in Java and can be run on any platform with a Java Runtime Environment e.g. Linux, MacOS and Windows. It has a simple command line interface for operation and runs rapidly. Alvis accepts inputs in the following formats: BLAST tabular, SAM files from BWA [17] and other aligners, PAF files from minimap2 [11], MUMmer’s .coords and .tiling files [6], and PSL files from BLAT [18]. Additionally, the software has been designed to allow easy extension to other formats. Diagrams can be output in either SVG or TeX formats. In the latter case, a LaTeX compiler is required to produce a PDF file.

The user can choose to filter alignments to remove noise. This is achieved by discarding alignments that are, by default, less than 1% of the query length (this value may be changed by the user). Alvis can be run from the command line using the Jar file provided in the distribution. For example, to create a coverage map from a minimap2 file:

Java -jar Alvis.jar -inputfmt paf -outputfmt tex -type coverageMap -coverageType long -in alignments.paf -outdir output -out outprefix

### Alvis diagrams

#### Alignment diagram

The alignment diagram (Fig. 1a) groups alignments by their target sequence, represented by a red bar at the top of the diagram. Each alignment is displayed as a rectangle underneath the bar, in line with the alignment position on the target. For a given target sequence, each alignment is ordered by the query ID and, for queries producing multiple alignments, by the start position of the alignment on the target. The length of each query sequence, and the position of the alignment on the query sequence, is indicated by a line parallel to the target bar and through the alignment rectangle. An example use case is to visualise a set of assembled contigs or long reads that align to a given chromosome.

#### Contig alignment diagram

The contig alignment diagram (Fig. 1b) groups all alignments by their query sequence, and for each query, displays the ten longest alignments inside a rectangle representing the query sequence. These alignments are colour coded by target sequence and shaded to indicate position and orientation. With this diagram, it is possible to view an assembled contig or long read to understand how discontinuously it maps to a target sequence and if it is chimeric. Chimeric query sequences are indicated with a ‘C’ next to the rectangle representing them. If the -chimeras option is specified, this diagram displays only query sequences which are chimeric, and in this case the ‘C’ is not displayed.

#### Coverage map diagram

The coverage map diagram (Fig. 1c) builds a coverage representation of a set of target sequences by dividing each into positional bins and incrementing the bin count for each query sequence that aligns to that bin. In the case of a query sequence with overlapping alignments, the largest alignment is chosen, and overlapping alignments are discarded. For each target sequence, a heat map image is produced with each bin represented by a pixel width. This image can be chosen to be either a square which wraps around, or a long bar (Fig. 1c). Since each target sequence may have a different length, the pixel size is adjusted so that the heat map size is constant. An early version of Alvis was used to generate resistance gene coverage maps in [19].

#### Genome coverage diagram

The genome coverage diagram (Fig. 1d) builds up a coverage representation in the same manner as the coverage map diagram. However, instead of producing individual target heatmaps, a single heatmap is produced which shows the coverage of every target sequence, in order, with a constant pixel size. This allows, for example, production of a single coverage map which shows the coverage of all chromosomes of a species by an assembly.

### Chimera detection

As described above, the Contig Alignment diagram will automatically highlight query sequences that are potentially chimeric. Chimeric sequences are chosen by examining the arrangement of target alignments along each query sequence. The criteria for a query sequence to be highlighted as chimeric are that the query sequence must have exactly two non-overlapping alignments to either different target sequences, or different sections of the same target sequence. Furthermore, these two alignments must together cover 90% of the query sequence, and the length of each alignment must be at least 10% of the length of the query sequence (see Fig. 3 for some examples). These default values may be adjusted by the user.

**Fig. 2:**
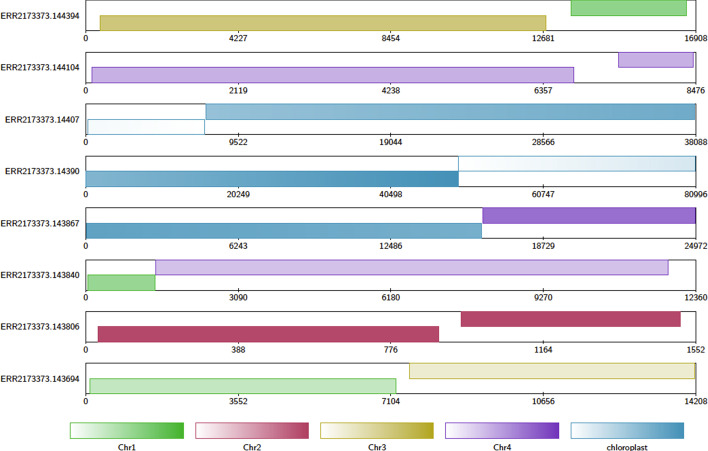
A contig alignment diagram of chimeric *A. thaliana* reads. Note that the read ERR2173373.14390 has two alignments, one to each end of the chloroplast reference sequence. Since chloroplast DNA is usually circular, this may be a relic of using linear sequence to represent circular DNA, and not a true chimera. Figure generated with Alvis v1.1

**Fig. 3:**
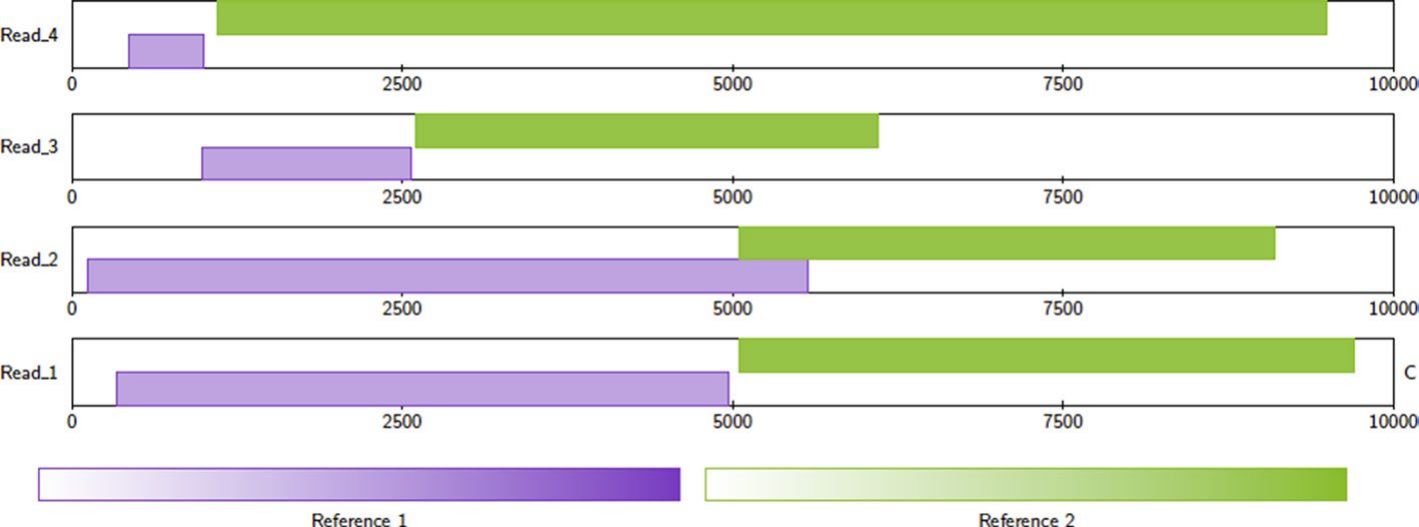
Example alignments. Read 1 is classified as chimeric. Read 2 is not classified as chimeric because the alignments overlap. This case is ambiguous—if the overlap is small, or there is sequence shared between Reference 1 and Reference 2, then this read could be chimeric. Read 3 is not classified as chimeric because the coverage of the alignments is too small. Read 4 is not classified as chimeric because the alignment to Reference 1 is too small. Figure generated with Alvis v1.2

When the -printChimeras option is specified, Alvis will output a separate plain text file containing the IDs of potentially chimeric reads or contigs, along with an approximate position of the join. Each line of this file represents a chimeric query sequence, where the first column is the query sequence name, the second column is the approximate position of the join (midway between the two alignments), and the third and fourth columns are the names of the target sequences for each alignment.

### Software architecture

The software has been designed in a modular fashion to enable easy future extension. Abstractly, there are three different components; Alignments, Drawers, and Diagrams.

#### Alignments

Each alignment format is represented by a class implementing either the Alignment or DetailedAlignment interface, depending on the data that is available in that format. The DetailedAlignment class extends the Alignment class. Another class representing the alignment file parses the file and creates an array of Alignment or DetailedAlignment objects; this class must implement either the AlignmentFile interface, or the DetailedAlignmentFile interface (which extends the AlignmentFile interface). Thus implementation of a new alignment format requires just two short classes to be created.

#### Drawers

Similarly, the notion of an output format has been abstracted through the Drawer interface. Currently two classes implement the Drawer interface; one for TeX output, which uses the Tikz package, and one for SVG output. Further output formats can be implemented through writing a class which implements the Drawer interface. This interface contains functions for basic drawing operations, such as drawing a line between two points, and drawing text.

#### Diagrams

Alvis is capable of drawing four diagrams, as discussed in the previous section. Each diagram takes an object implementing either the AlignmentFile or DetailedAlignmentFile interfaces, and creates an instance of a Drawer to output the diagram. The Alignment and Contig Alignment diagrams require an object implementing the DetailedAlignmentFile interface, while the Coverage Map and Genome Coverage diagrams require an object implementing the AlignmentFile interface, which, thanks to inheritance, also includes objects implementing the DetailedAlignmentFile interface.

## Results

To demonstrate the efficacy of Alvis’ chimera detection function, we downloaded and assembled two sets of nanopore reads, and compared chimera detection with YACRD. The first of these sets was sequenced from *Arabidopsis thaliana* accession KBS-Mac-74 [20], and the second was the Rel3 nanopore Human dataset, taken from [21]. For each read set we mapped reads to the respective reference genome (TAIR10 [22] for *A. thaliana*, and GRCh38.p13 [23] for human) using minimap2, creating two large PAF files to be used by Alvis. For YACRD, we used minimap2 to align each read set to itself, to create two PAF files of overlaps.

Using YACRD with default parameters, we found only a single chimera for the human dataset, and none for the *A. thaliana* dataset.

Using Alvis with default parameters, we parsed the read-to-reference alignment files, which highlighted those reads that it believed to be chimeric. Figure 2 shows contig alginment diagrams for a handful of chimeric contigs taken from the *A. thaliana* read set. In this dataset we found a total of 2817 out of 300,071 reads meeting Alvis’ chimera requirements. Of these, 264 reads had alignments to the same circular reference sequence (either mitochondria or chloroplast) and so were ignored, leaving 2553 potential chimeric reads.

**Table 2:** Contig statistics for Flye assemblies of *A. thaliana* reads before and after chimera splitting using Alvis’ chimera detection

Assembly	Before	After
Number of contigs	282	250
Total bases	131,621,845	130,854,472
Mean contig length	466,744	523,418
Shortest	435	547
Longest	16,289,205	15,366,049
N50	12,353,468	13,568,072
N90	357,247	693,429

**Table 3:** Contig statistics for Flye assemblies of human reads before and after chimera splitting using Alvis’ chimera detection

Assembly	Before	After
Number of contigs	247	251
Total bases	8,295,215	8,945,628
Mean contig length	33,583	35,640
Shortest	522	520
Longest	135,585	135,924
N50	50,325	50,524
N90	20,595	23,079

We then created a new read set, by copying all the original reads, and splitting each of the 2553 potentially chimeric reads at the join position given by Alvis in the -printChimeras file. A python script to do this automatically is available in the Alvis distribution. Both the new read set and the original read set were assembled using Flye [24]. A significant improvement in the contiguity of the assembly from the split reads can be seen in Table 2.

For the human dataset, we found a total of 1750 out of 658,224 chimeric reads, of which 90 had alignments to the mitochondrial sequence in the reference genome, so these were ignored. This left 1660 chimeric reads in total. As before, two assemblies were performed using Flye; one from the original read set, and one from the read set where chimeras were split. The contiguity statistics for these assemblies are presented in Table 3.

To assess any change in the correctness of the assemblies from splitting chimeric reads, we ran the dnadiff [6] program on each *A. thaliana* assembly against the TAIR10 reference assembly. Before splitting, 96.84% of bases aligned to the reference genome with 7205 1-to-1 alignments at an average identity of 97.31%. After splitting, 96.98% of bases aligned to the reference genome with 7182 1-to-1 alignments at an average identity of 97.32%. The assembly before splitting had 33,764 M-to-M alignments, with an average identity of 95.84%, while the assembly after splitting had 33,381 M-to-M alignments, with an average identity of 95.88%. Overall then, there was no significant change in the dnadiff results.

## Conclusions

Alvis provides the ability to quickly and simply visualise contig and read alignments in four diagram types. A wide variety of popular file formats are supported and a simple API provides for future extension. Diagrams can be output as vector images, providing high quality publication-ready figures. The ability to output to SVG could allow future integration with web applications. Crucially, Alvis can also be used to highlight potential chimeric reads and contigs. In the former case, splitting chimeric reads can lead to more contiguous assemblies with a higher N50, without any loss in correctness.

## Availability and requirements

**Project name:** Alvis

**Project home page:** https://github.com/SR-Martin/alvis

**Operating system:** Platform independent

**Programming language:** Java

**Other requirements:** Java Runtime Environment

**License:** GNU GPL v3

**Any restrictions to use by non-academics:** No

## Data Availability

The data used to generate the diagrams in Fig. 1 is available in the Alvis repository at https://github.com/SR-Martin/alvis. A detailed tutorial for creating these diagrams is available at https://alvis.readthedocs.io/en/latest/usage/example.html.

## Abbreviations

PAF: Pairwise mApping Format, a file format output by the tool minimap2
SAM: Sequence Alignment/Map, a file format output by the tool BWA and others
SVG: Scalable Vector Graphics, a vector image file format

## References

[1] Zerbino DR, Achuthan P (2018). Ensembl 2018. Nucleic Acids Res.

[2] Carver T, Harris SR, Berriman M (2011). Artemis: an integrated platform for visualization and analysis of high-throughput sequence-based experimental data. Bioinformatics.

[3] Darling ACE, Mau B, Blattner FR (2004). Mauve: Multiple alignment of conserved genomic sequence with rearrangements. Genome Res.

[4] Mikheenko A, Valin G, Prjibelski A (2016). Icarus: visualizer for de novo assembly evaluation. Bioinformatics.

[5] Gurevich A, Saveliev V, Vyahhi N (2013). Quast: quality assessment tool for genome assemblies. Bioinformatics.

[6] Kurtz S, Phillippy A, Delcher AL (2004). Versatile and open software for comparing large genomes. Genome Biol.

[7] Krzywinski M (2009). Circos: an information aesthetic for comparative genomics. Genome Res.

[8] Schnable PS (2009). The b73 maize genome: Complexity, diversity and dynamics. Science.

[9] Boratyn GM, Camacho C, Cooper PS (2013). Blast: a more efficient report with usability improvements. Nucleic Acids Res.

[10] Leggett RM, Clark MD (2017). A world of opportunities with nanopore sequencing. J Exp Bot.

[11] Li H (2018). Minimap2: pairwise alignment for nucleotide sequences. Bioinformatics.

[12] White W, Pellefigues C, Ronchese F, *et al*. Investigation of chimeric reads using the minion. f1000Research 6, 631;2017. 10.12688/f1000research.11547.2 10.12688/f1000research.11547.1 PMC5600009 28928943

[13] LaPierre N, Egan R, Wang W (2019). De novo nanopore read quality improvement using deep learning. BMC Bioinform.

[14] Marijon P, Chikhi R, Varré JS (2020). yacrd and fpa: upstream tools for long-read genome assembly. Bioinformatics.

[15] Edgar RC, Haas BJ, Clemente JC (2011). Uchime improves sensitivity and speed of chimera detection. Bioinformatics.

[16] Haas BJ, Gevers D, Ashlee ME (2011). Chimeric 16s rrna sequence formation and detection in sanger and 454-pyrosequenced pcr amplicons. Genome Res.

[17] Li H, Durbin R (2009). Fast and accurate short read alignment with burrows-wheeler transform. Bioinformatics.

[18] Kent JW (2002). Blat - the blast-like alignment tool. Genome Res.

[19] Jupe F (2013). Resistance gene enrichment sequencing (renseq) enables reannotation of the nb-lrr gene family from sequenced plant genomes and rapid mapping of resistance loci in segregating populations. Plant J.

[20] Michael TP, Jupe F, Bemm F (2018). High contiguity *Arabidopsis thaliana* genome assembly with a single nanopore flow cell. Nat Commun.

[21] Jain M, Koren S (2018). Nanopore sequencing and assembly of a human genome with ultra-long reads. Nat Biotechnol.

[22] Lamesch P, Berardini TZ, Donghui L, Swarbreck D (2012). The Arabidopsis information resource (tair): improved gene annotation and new tools. Nucleic Acids Res.

[23] Schneider VA, Graves-Lindsay T, Howe K, *et al*. Evaluation of grch38 and de novo haploid genome assemblies demonstrates the enduring quality of the reference assembly. 2016. 10.1101/gr.213611.116 10.1101/gr.213611.116 PMC5411779 28396521

[24] Kolmogorov M, Yuan J, Lin Y, Pevzner P (2019). Assembly of long error-prone reads using repeat graphs. Nat Biotechnol.

